# Disparities between two possible thresholds for frequent contacts to a Norwegian emergency medical communication centre: ≥5 contacts in one month vs. ≥12 contacts in three months

**DOI:** 10.1186/s12873-025-01333-6

**Published:** 2025-08-29

**Authors:** Astrid K. V. Harring, Magnus Hjortdahl, Kristin Häikiö, Trine M. Jørgensen

**Affiliations:** 1 Oslo Metropolitan University, PB 4, St. Olavs plass, Oslo, 0130 Norway; 2 Department of Community Medicine, Norwegian Centre for Rural Medicine, UiT The Arctic University of Norway, Tromsø, Norway; 3 Division of Prehospital Services, Oslo University Hospital, Oslo, Norway

**Keywords:** Emergency medical communication centre, Emergency medical services, Frequent caller, Prehospital

## Abstract

**Background:**

Frequent callers are described as a challenge for Emergency Medical Services (EMS) across international contexts, but the terminology, definitions, and frequency vary. Until 2023, the UK’s Frequent Caller National Network (FreCaNN) definition was ≥ 5 incidents in one month or ≥ 12 incidents in three months. In this study we applied that definition to compare the two possible thresholds independently on data from Norway’s largest Emergency Medical Communication Centre (EMCC).

**Methods:**

A retrospective cross-sectional database review of all patients who met the former FreCaNN definition of frequent contacts to the medical emergency number 1-1-3 at Oslo EMCC between 1. January 2017 and 31. December 2022, also including all ages, and instances where others called on behalf of a patient.

**Results:**

A total of 19.559 unique identities met the inclusion criteria. Of them, 16.518 patients accumulated 130.814 contacts to the EMCC relating to individual episodes 5 or more times in one month, but fewer than 12 in three months. For the group with 12 or more contacts in three months, 3.041. patients accumulated 137.909 contacts. Almost all those with ≥ 12 contacts within three-months also met the ≥ 5 contacts in one month criterion. Those who had ≥ 12 contacts in three months tended to be significantly younger (median age: 58 vs. 70) and more likely to make the call themselves (64% vs. 32%). Regarding chief complaints, they had a higher frequency of “unidentified problems” (34% vs. 22%), “mental health problems” (20% vs. 8.2%), and “intoxication/OD” (7.2% vs. 4.4%) and were less likely to be prioritized as urgent (54% vs. 82%). An ambulance was less often dispatched (38% vs. 69%), and they were less often taken to either a doctor (17% vs. 25%) or to a hospital (13% vs. 32%) (all *p* < .001).

**Conclusion:**

These results show disparities and indicate that there are two distinct subgroups within the former FreCaNN definition. In the Norwegian context focusing on the ‘12 contacts in three months’ component allows for improved specificity and a more manageable number of individuals, for further research to identify effective targeted clinical measures to improve patient care.

**Clinical trial number:**

Not applicable.

**Supplementary Information:**

The online version contains supplementary material available at 10.1186/s12873-025-01333-6.

## Introduction

The Frequent Caller National Network (FreCaNN) is a UK organisation based on collaboration between several of their ambulance trusts. Up until 2022, they defined a frequent caller as an individual aged 18 years or over, who makes 5 or more emergency calls related to individual episodes of care in one month or 12 or more emergency calls related to individual episodes of care in three months [1]. The most recent definition uses a data backed threshold of five or more incidents [2] and have disregarded the quarterly definition [3]. In a recent scoping review of the international literature, we found that what could be considered “frequent” range from 4 to 120 calls or 3 to 10 ambulance responses, annually [4], highlighting the necessity of a consistent definition of frequent callers to, and users of, the Emergency Medical Services (EMS) [4, 5].

Social factors such as self-reported loneliness, poverty, and a low quality of life are associated with frequent callers [6]. The majority have multiple and complex reasons for calling, including medical needs, acute or chronic mental health conditions and older age [7]. Mahmuda et al. [8] has identified primary (falls, chronic disease, etc.) and secondary factors (no social support, inability to access services, etc.) for why frequent EMS users call. Common medical conditions, such as uncontrolled hypertension and hypoglycaemia, can trigger frequent emergency calls, and medication nonadherence might contribute to the exacerbation of existing medical conditions [9]. Frequent callers are often users of, and in contact with, multiple healthcare services [10, 11]. Thus, many ambulance services have employed a multi-disciplinary ‘case management’ model through cross-service partnerships. Such teams, aimed to address callers’ clinical, social or emotional needs typically include clinicians, health service managers, voluntary sector representatives and the police [12].

A recent study from the Emergency Medical Communication Centre (EMCC) in Bergen, Norway compared frequent to non-frequent callers [13]. They used a threshold of *≥ 5* contacts over 12 consecutive months and found that frequent callers had a lower proportion of calls with an acute priority, a lower rate of ambulance dispatch, and that “mental health problems/suicide” was their most common contact criteria. Due to the higher proportion of “no EMS response” or verbal referral to the local out-of-hours clinic (OOHC), many of these contacts could be managed elsewhere in the healthcare system [13]. They reported that 3.2% of individuals were frequent callers, and that those frequent callers accounted for nearly one in four calls. The Bergen study included all types of callers without explicitly stating it, corresponding to our previous results, which demonstrates that, in addition to patients who frequently call the EMCC themselves, others also frequently call on behalf of the patients [14]. To capture all types of callers, we introduced the term frequent emergency contacts, which we found to constitute 13% of all EMCC contacts [14]. The Bergen study concludes that there is a need to identify frequent callers and to offer more appropriate services, that align with their needs [13].

In this study, we evaluate the two parts of the former FreCaNN definition of “*≥5 contacts in one month* and *≥ 12 contacts in three months*” separately using data from Norway’s largest EMCC, comparing characteristics such as demographics, type of caller, perceived urgency and allocated response to explore possible differences.

## Method

**Fig. 1:**
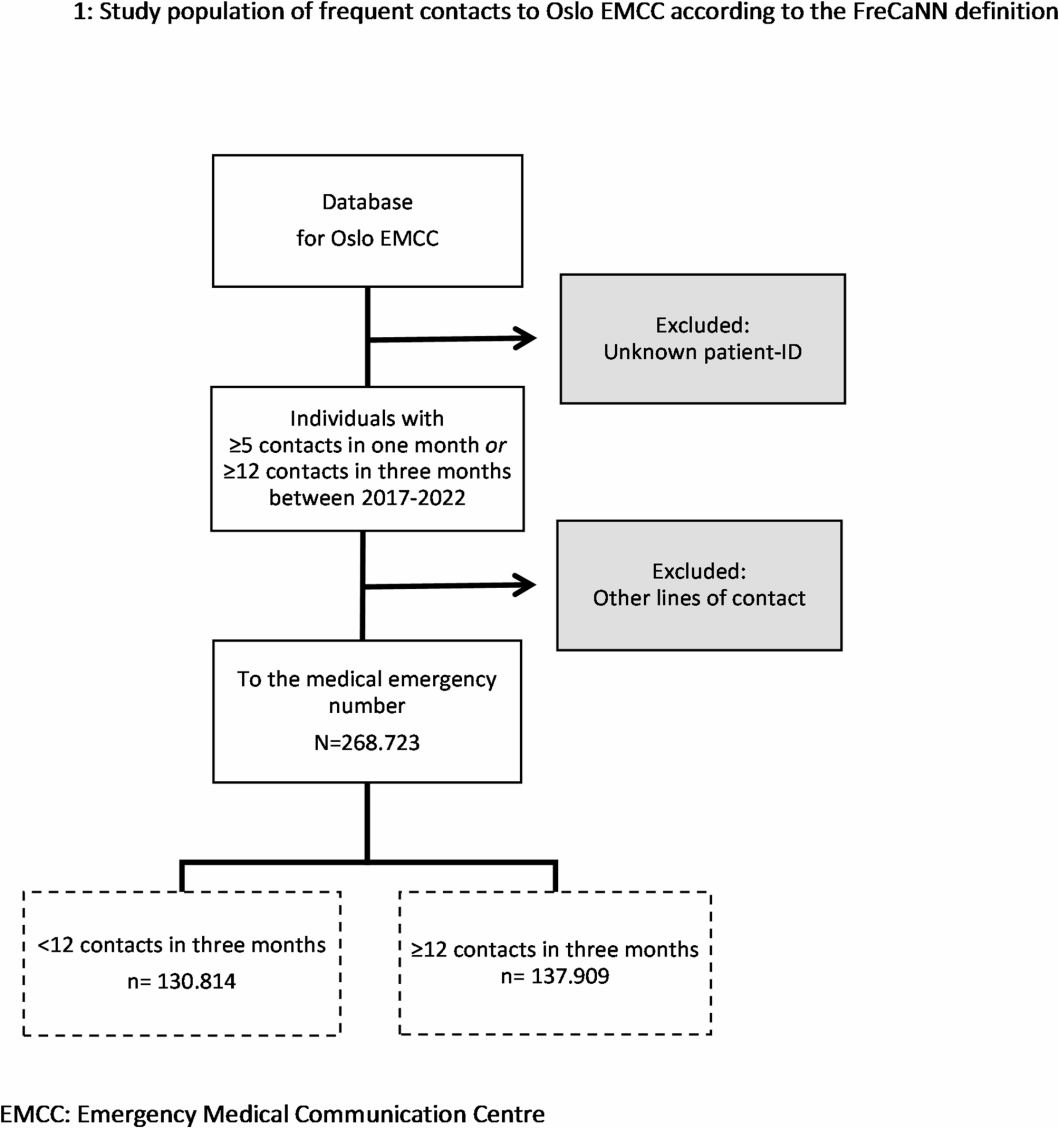
Study population of frequent contacts to Oslo EMCC

In this retrospective cross-sectional database review, we included medical emergency calls for patients registered with a Norwegian personal identity number, with ≥ 5 contacts in one month, or ≥ 12 in 3 months to Oslo EMCC between 1. January 2017 and 31. December 2022 (Fig. 1). We included patients of all ages and instances where others called on behalf of the patient.

### Setting

The healthcare system in Norway is government founded and divided into primary and specialist healthcare. All citizens have a general practitioner (GP) that is the main point of contact. Primary healthcare also includes OOHCs, home-based care, nursing homes and assisted living institutions. Specialist healthcare includes all hospitals, medical helicopters and ambulance services. The two “systems” are combined by the Regulation on Requirements for Emergency Medical Services Outside Hospitals, and a comprehensive description of the Norwegian EMS [15] has recently been published.

Norway has separate emergency numbers; one for the police (1–1–2), one for the fire department (1-1-0) and one for medical emergencies (1-1-3). For urgent medical care the OOHC has a national number (116–117). In all the Norwegian EMCCs the medical telephone operator is either a registered nurse, emergency medical technician (EMT) or paramedic. In all calls, the EMCC operator uses the Norwegian Index for Emergency Medical assistance [16] for decision support, along with their clinical reasoning. The third and fourth version (used in the data gathering period) consisted of 39 chief complaints (many of which are shown in Table 1). For each of the chief complaints, there are several sub-criterions recommending what the index deems as an appropriate priority:

1. *Acute* - for life threatening events, acute chest pain, stroke, or overdose with laboured breathing.
2. *Urgent* - for unclear, potentially serious or painful conditions such as dislocations or discomfort at the side of the chest. Contacts in this category can be transferred to OOHC, especially for ambulatory patients or where the patient likely could benefit from being assessed by a doctor at home.
3. *Non-urgent* – for minor or long-lasting symptoms such as short and sharp pains in the chest, psychosocial issue or transport to planned procedures or from hospital to home.

A full list of the sub-criterions for “unidentified problem” are provided in Supplementary Table 1.

The operator is at liberty to determine if an ambulance should respond to the call or not. For cases where no EMS resource is dispatched, the call can either be referred to the OOHC for further evaluation or resolved by the EMCC operator (similar to the “Hear & Treat” in the UK [1]).

When an ambulance resource is dispatched the ambulance clinicians (EMTs or paramedics) assess the patient and decide whether the patient should be transported to a GP/OOHC, hospital, or not transported at all. The ambulance clinicians can admit patients directly to the hospitals’ emergency department if deemed necessary. If the need for hospitalization is non-urgent or unclear, the patient is brought to their GP/OOHC for further evaluation. If the call regards a need for psychiatric hospitalization, a GP/OOHC doctor must assess and admit the patient before the ambulance continues their conveyance.

In some ambulance services there are designated white “patient transport vehicles” for non-urgent transfers, a few have singe-response units, but none have a community paramedic service or paramedic prescribers.

### Data collection

The dataset is provided by Oslo EMCC, the largest EMCC in Norway. The centre is located at Oslo University Hospital and handles 25% of all the medical emergency calls in Norway. It encompasses the capital and surrounding municipalities, both urban and rural, and covers approximately 1.7 million inhabitants, receiving approximately 248,000 medical emergency calls annually [17]. During the six-year period, Oslo EMCC registered 2.149.400 contacts, of which 1.525.564 were emergency calls.

As shown in Fig. 1, the identification and inclusion of individuals occur prior to the exclusion of other lines of contact, as most non-urgent transport requests for healthcare personnel are made through a separate telephone number or web-order system. While these contacts are exempt from this study, a patient will be included in the dataset if they have five or more contacts in total in one month or twelve or more in three months.

The data were collected from the Emergency Medical Information System (AMIS). The Patient ID is linked and gathered form the population registry; the operator can search by social security number, full name or date of birth and parts of the name. The dataset was checked for duplicates, anonymised, and assigned a study ID-number to each unique identity prior to being exported to their secure research server for data handling.

### Variables

The variables and sources in the dataset are listed in Supplementary Table 2. To account for the month of February in nonleap years, the FreCaNN definition of one month was operationalized to 28 days, and three months were operationalized to 90 days in the data subtraction. The system screened all AMIS data, calculated whether the criteria were met within the observation period, and, if so, collected data regarding that patient, from the first call that was included when they met the criteria, throughout the entire observation period.

The transport options in this dataset are “no transport”, “transport to GP/OOHC” or “transport to hospital”.

Because of the need for further examinations or doctor referrals for non-urgent, unclear or psychiatric admissions a patient can be present at two transport destinations in the dataset for the same contact. If the ambulance clinicians decide not to transport, other arrangements can have been made, or they have deemed that there was no need for transport or referral (similar to the “See & Treat” in the UK [1]).

### Groups

Given that the definition comprises two distinct components, we hypothesized that it may encompass two quite different populations that are combined within one definition. Here we aim to study the two parts separately, comparing the two, thus we divided the dataset into two groups:

- those who had few contacts, i.e., < 12 contacts in three months.
  (but ≥ 5 contacts in one month)

- those who had many contacts, i.e., ≥ 12 contacts in three months.

For short, “few (< 12)” and “many (≥ 12)” are used to differentiate the two groups.

### Statistics

Descriptive statistics are presented as frequencies and percentages for categorical variables and medians with quartiles (Q) for continuous variables. To allow for future meta-analyses we have also included the mean age with standard deviation (SD). Chi-square tests were used to compare categorical variables and independent Mann-Whitney U test for continuous variables. The significance level was set to a P value of ≤ 0.05. Statistical analyses were performed via SPSS Version 29.0.0.0 (IBM Corporation). Figure 4 and the corresponding Clopper–Pearson exact confidence interval (CI) for differences in percentages were generated in Spyder version 5.4.1 (Scientific PYthon Development EnviRoment), using Matplotlib.

### Ethics

We submitted a preapproval application to the Regional Medical Ethics Committee, which considered the study to be regarded as quality assurance and improvement and thus to be outside of its scope, according to the Health Research Act (nr. 263844). The Norwegian Directory of Health waived the requirement for patient consent (nr. 23/7305-2). The Data Protection Officer at Oslo University Hospital approved the study and provided access to a secure research server for data handling (nr. 21/14225).

## Results

**Fig. 2:**
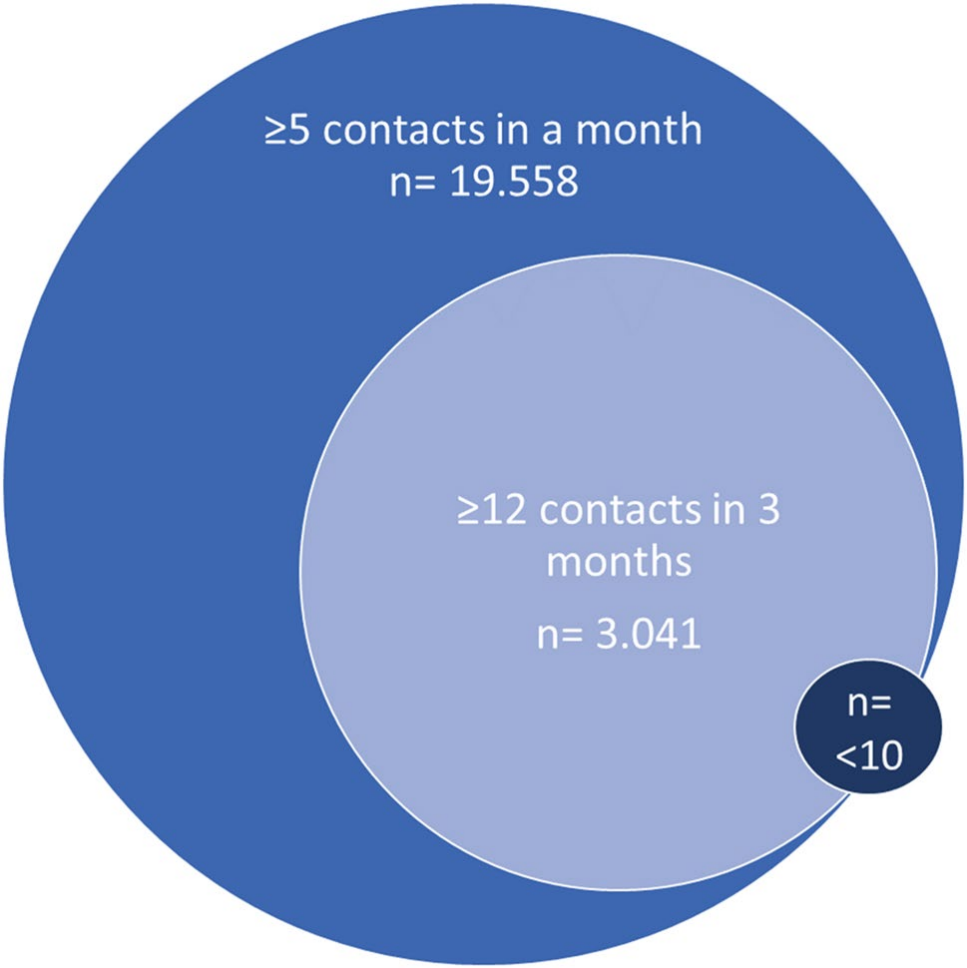
Number of individuals meeting the different parts of the FreCaNN definition. Almost all who were covered by ≥ 12 contacts in three months, were also included in the ≥ 5 times-in-a-month criterion

In total, 268.723 contacts regarding 19.559 unique identities meet the FreCaNN definition in the six-year period, when including all ages and types of callers. Since nearly all patients who had 12 or more contacts in three months also had 5 or more contacts in one month, those who had 12 or more contacts in three months can be viewed as a subgroup (Fig. 2). Under ten patients had four contacts each month for three or more consecutive months, thus only meeting the latter part of the criterion.

**Fig. 3:**
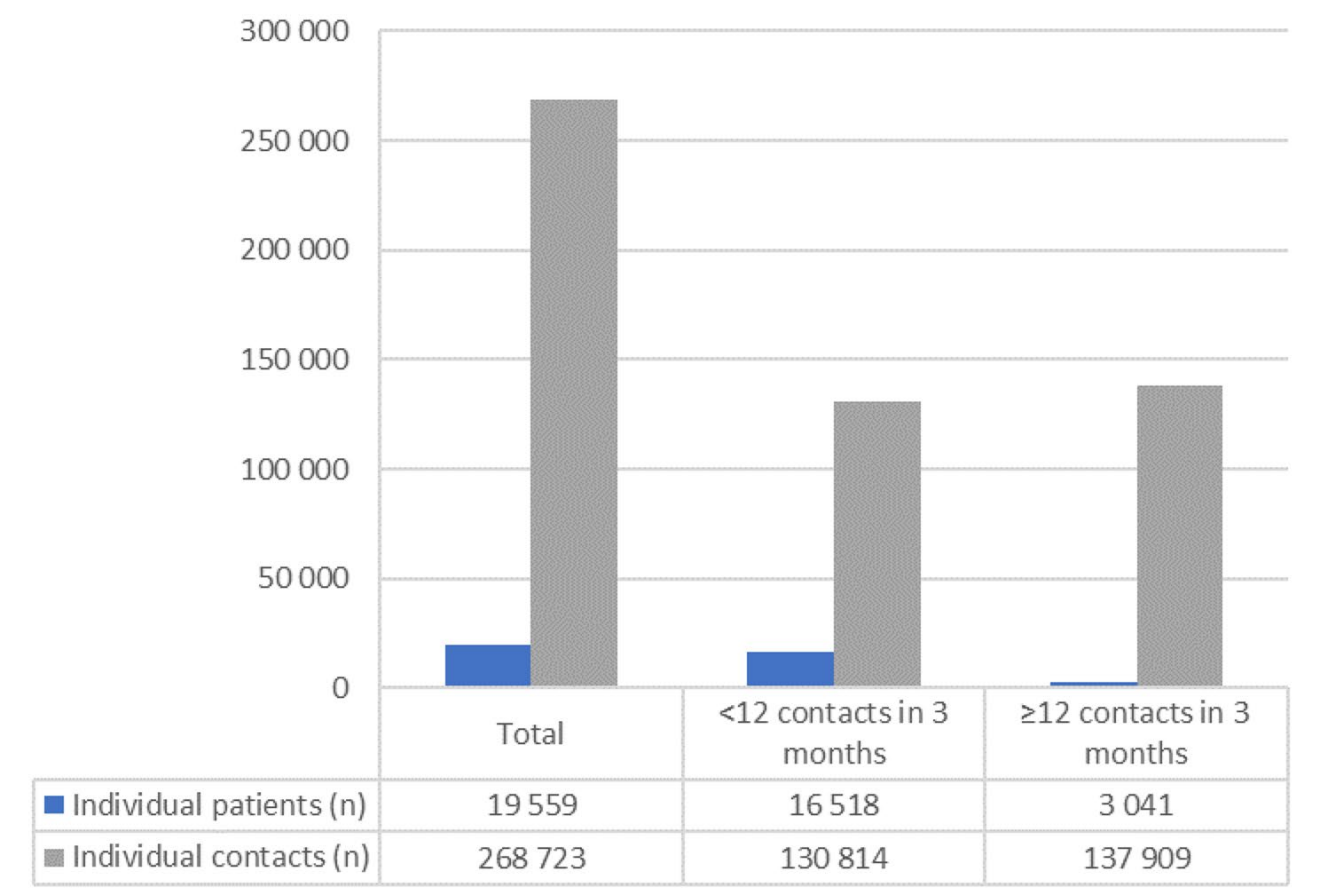
Comparing the number of individual patients to the number of contacts on behalf of the patient i.e. individual contacts (*N* = 268.723)

As shown in Fig. 3, individual callers who had few (< 12) contacts accounted for 130.814 contacts (median per patient: 6, Q1: 3; Q3: 10), whereas the ≥ 12 group accounted for 137.909 contacts (median per patient: 21, Q1: 9; Q3: 44,5). This means that those who had many (≥ 12) contacts comprised 16% of the individuals, yet accounted for 51% of the contacts.

A total of 268.723 contacts were distributed across 19.559 patients, resulting in an average of 14 contacts per patient. Distributed over a six-year period, this equates to approximately 2.3 contacts per patient per year. When stratified by contact frequency, patients with few (< 12) contacts, averaged 7.9 contacts per patient (SD: 7.6), equal to 1.3 contacts per patient per year, whereas those who had many (≥ 12) contacts had, on average 45 contacts per patient (SD: 171), equal to 7.5 contacts per patient per year.

Those who had few (< 12, but ≥ 5) contacts were statistically significantly older than those who had many (≥ 12) contacts (Table 1). The median age was 70 years (Q1: 49; Q3: 82), compared to 58 years (Q1: 44; Q3: 70, *p* < .001) (mean: 65 years, SD: 22; vs. 57 years, SD: 18). The number of patients under the age of 18 years was similar between the two groups (1.4% vs. 1.2%).

Regarding gender, we found no difference at the individual patient level (both 48% female). However, as shown in Table 1, there was a significant difference in the number of contacts.

As presented in Table 1, there are three main types of callers (patient, next-of-kin and healthcare personnel) that constitute approximately 80% of the contacts, but the distribution differs largely between the two groups. For those who encounter the EMCC many (≥ 12) times, the calls are more often made by the patient themselves (64% vs. 32% % for < 12) and proportionately less by others, on behalf of or regarding the patient.

There is a large variety of chief complaints, indicating a complex and heterogeneous group (Table 1). When comparing the groups, there is a notable increase in “unidentified problem”, “mental health problem” and “intoxication/overdose” for those with many (≥ 12) contacts, whereas all other complaints decreased.

**Table 1:** Comparing type of caller, chief complaint, priority and response for each contact to the EMCC (*N* = 268 723)

	Few contacts (< 12 in 3 months)		Many contacts (≥ 12 in 3 months)		χ²	Significance
	Frequency	%	Frequency	%		
**Total number of contacts** ($\boldsymbol{\mathrm{\tilde x}}$)	130 814 (6)	-	137 909 (21)	-		
Age, median (IQR)	70 (33)	-	58 (26)	-		**< 0.001**
Female	63 733	49	72 828	53	*df 1 = 449*	**< 0.001**
**Type of caller**					*df 13 = 32 944*	**< 0.001**
Patient	41 332	32	88 368	64		
Next of kin	32 741	25	13 558	10		
Healthcare personnel	30 652	23	13 729	10		
The public	11 190	8,6	10 665	7,7		
OOHC	3 989	3,0	1 892	1,4		
Doctor	2 623	2,0	1 158	0,8		
Police	2 104	1,6	2 094	1,5		
Neighbour	1 884	1,4	1 229	0,9		
Other EMCC	317	0,2	244	0,2		
Fire department	112	0,1	91	0,1		
N/A	3 870	3,1	4 881	3,4		
**Chief complaint**					*df 39 = 22 157*	**< 0.001**
Unidentified problem	28 776	22	47 240	34		
Breathing problems	14 153	11	10 343	8		
Chest pain	13 518	10	9 818	7,1		
Mental health problems	10 789	8,2	27 194	20		
Abdominal/ back pain	9 424	7,2	7 441	5,4		
Transport	8 696	6,6	3 934	2,9		
Altered level of consciousness*	7 133	5,5	2 815	2,0		
Intoxication/overdose	5 767	4,4	7 320	7,2		
Minor injuries	5 359	4,1	2 567	1,9		
Major injuries	4 576	3,5	2 060	1,5		
Seizures	2 445	1,9	1 695	1,2		
Unresponsive, adult	2 349	1,8	1 327	1,0		
Urinary tract problems	2 221	1,7	1 515	1,1		
Bleeding, non-traumatic	1 905	1,5	688	0,5		
Fever	1 882	1,4	679	0,5		
Headache	1 862	1,4	1 474	1,1		
Diabetes	1 275	1	554	0,4		
Chief complaints < 1%	4 987	5,0	4 534	2,8		
N/A	3 697	2,8	4711	3,4		
**Priority**					*df 2 = 26 756*	**< 0.001**
Priority 1, acute	45 895	35	25 262	18		
Priority 2, urgent	57 913	44	43 918	32		
Priority 3, non-urgent	23 309	18	64 018	46		
N/A	3 697	2,8	4 711	3,4		
**Response**						
Ambulance not dispatched	40 853	31	85 088	62	*df 1 = 25 028*	**< 0.001**
Ambulance dispatched	89 961	69	52 821	38		
Ambulance transport to GP/OOHC	32 925	25	23 222	17	*df 1 = 2 819*	**< 0.001**
Ambulance transport hospital	41 894	32	18 223	13	*df 1 = 13 680*	**< 0.001**

*Include confusion, dizziness, and stroke symptoms

Chief complaints < 1%: Chief complaints with a frequency of less than 1% were combined into one category

EMCC: Emergency Medical Communication Centre; GP: General practitioner; OOHC: out-of-hours clinic

X̃: median (per patient), IQR: Inter quartile range, df: degrees of freedom

χ²: Pearson’s chi-squared test, used to compare categorical variables between the two groups. No post-hoc analysis were performed on sub-variables

**Fig. 4:**
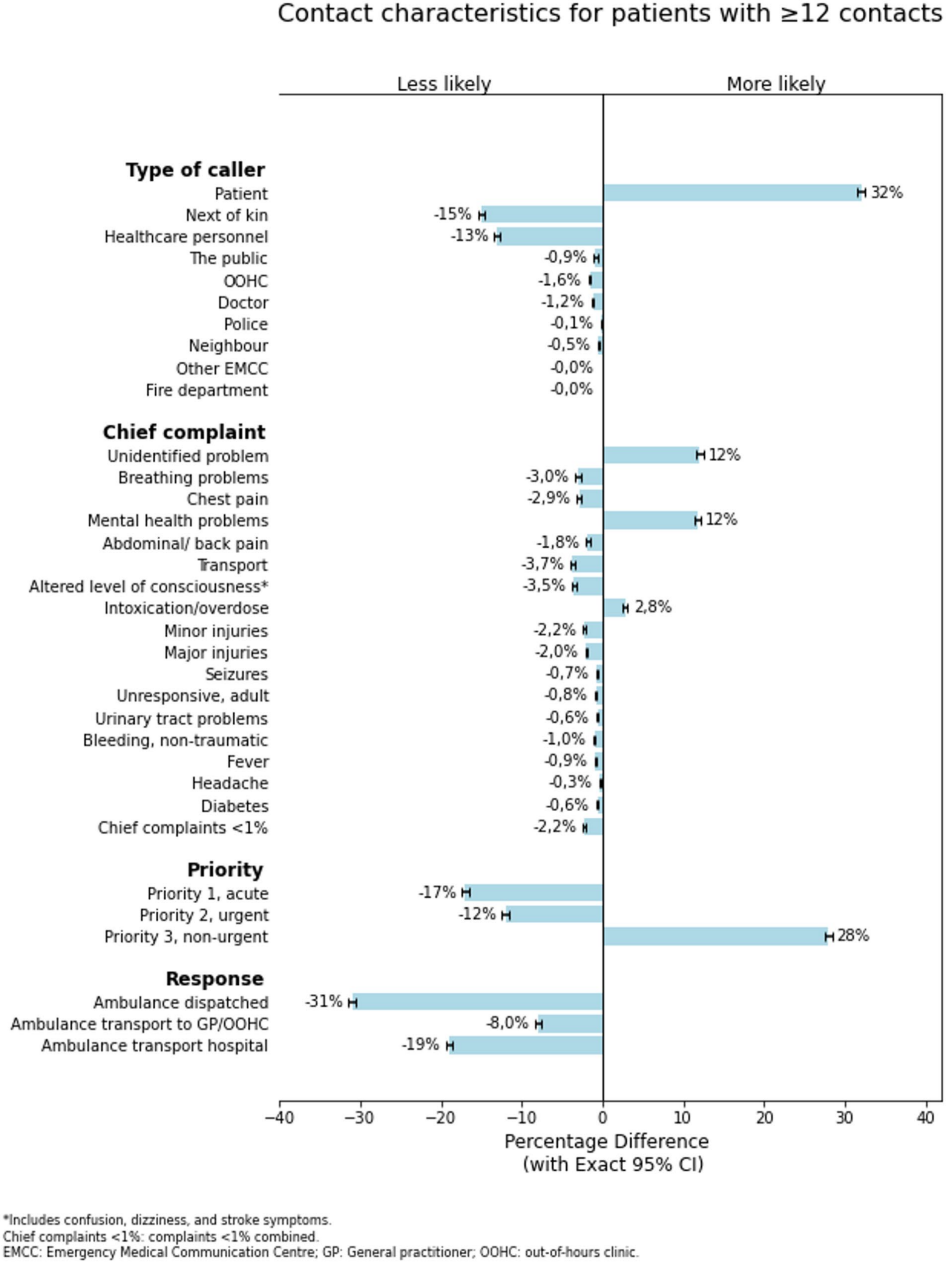
Contact characteristics for patients with many (≥ 12) contacts compared to those with few (< 12) (*N* = 268.723)

As seen in Fig. 4, differences in emergency call characteristics of each of the contacts in the two groups were compared (values are provided in Supplementary Table 3). Those who were in contact with the EMCC ≥ 12 times were more likely to be assigned a “non-urgent” priority, and an ambulance was less often dispatched. As seen in Table 1, the proportions of Priority 1 contacts to hospital admissions by the EMS are nearly identical in both groups (35% vs. 32% and 18% vs. 13%, respectively). Regardless of group (< 12 or ≥ 12), there was a significant association between priority and EMS dispatch (*df 2 = 139.759, p < .001*), and priority and hospital admission (*df 2 = 48.741, p < .001*). Both groups combined, the overall hospitalization rate was 23%. Of those, 59% (*n* = 35.476) was dispatched as priority 1; however, some (4.3%) of the hospital admissions the contact were assigned priority 3 (non-urgent). Notably, there were also 36.734 fewer EMS dispatches than non-urgent priorities, suggesting that some urgent (and 535 acute) cases have been resolved without dispatching an ambulance.

## Discussion

Frequent callers are known to be a heterogeneous group [5, 7, 18]. Our results suggest that, while they remain heterogeneous overall, they become more homogeneous when divided into the two components of the previous FreCaNN definition: ≥5 individual episodes of care in one month and ≥ 12 episodes in three months, as we found the groups to differ in terms of age, gender, caller type, chief complaint, perceived urgency, and response.

### Disparities between the two thresholds

Those who had many contacts (≥ 12) were significantly younger and slightly more often female compared to the < 12 group. However, some outliers are likely to influence the results by having many contacts/calls. The literature has found mixed results regarding age and gender [2, 5, 13, 18]. Edwards et al. [7] and Scott et al. [18] also found a slightly lower percentage of males (45–46%) than females in their study on frequent caller profiling. Despite age and gender, analysed as group-level characteristics, having significant differences in statistical analysis, they cannot be used as individual predictors.

The calls were more likely to be made by themselves (64% vs. 32%) if they had been in contact with EMCC more than 12 times in three months. Figure 4 is inspired by Scott et al. [18], who compared the chief complaints of the top 100 most frequent callers to the population. We found the categories “mental health problems” and “unidentified problems” increased the most (both by 12%) between those with few (< 12) versus those with many (≥ 12) contacts. These findings coincide with the EMCC Bergen study, followed by intoxication/overdose, chest pain and breathing problems [13]. Similarly, Scott et al. [2] reported that chief complaints such as chest pain, psychiatric/suicide attempts and abdominal pain/problems were indicative of frequent use, defined as ≥ 5 incidents. We found that chest pain, breathing problems and abdominal pain decreased by 2–3% in the ≥ 12 group. Thus, there were still a high number (5–8%) of patients who reported these chief complaints among the ≥ 12 group. In interviews, Danish frequent callers explained that they called the emergency number in cases where they were not able to cope with their health conditions and difficult life circumstances themselves and further told that nobody else were there or able to help them out [19]. The latter is supported by Agarwal et al.’s findings [4], that many frequent callers live with moderate to extreme pain and discomfort, high degrees of anxiety and depression, and just under half were lonely.

In the chief complaint category “unidentified problems”, one finds the “psychosocial issue” criteria [16] (or see Supplementary Table 1). Edwards et al. [7] reported that the most common profile categories among frequent callers were medical needs, acute or chronic mental illness, being elderly, unmet social/personal care needs or having suicidal intentions or self-harm. Laursen et al. [19] found the concept of “struggling along” fitting to the experiences described by the frequent callers. In addition, their physical function has often been found to be impacted, as they report having mobility problems and difficulties conducting usual activities and self-care, impairing their access to ambulatory care and therefore calling the EMS more frequently [6, 8]. Similarly, Martinez et al. [9] found a lack of transportation as a barrier to medical care but also identified a need for assistance in navigating both medical and psychiatric care. If one is not able to access healthcare services, the EMS might be seen as their only or last resort if they have exhausted all other options, as in the example of “Peter” by Laursen et al. [19]. For those who had many contacts (≥ 12), the situations are often evaluated as less urgent, and an ambulance was dispatched less often than for those with fewer (< 12) contacts. This is supported by the EMCC Bergen study [13], who found a higher proportion of no response/verbal referral to the OOHC for frequent callers. Also, Scott et al. reported that frequent callers were assigned low-level responses in two separate studies [2, 18]. The appropriateness of the lower EMCC priority we discovered seems to be supported by the EMS response, as they were less likely to be taken to the GP, OOHC or transported to a hospital. This corresponds with the finding of Martinez et al., that EMS providers consider responses to chronic medical conditions and support with mental health symptoms important, but not as emergencies [9]. In our study, we find the EMCC operator’s ability to distinguish between situations that require an EMS response and those that can safely proceed without one, to be reflected in the high number of contacts with “no dispatch” (see Table 1).

### The ≥ 5 in one month criterion seems overly sensitive for the Norwegian context

A part of the reason for why patients with less than 12 times in three months more often had an ambulance dispatched and more often were taken to a GP, OOHC or hospital might be related to them being significantly older. Martinez et al. investigated older adult frequent 9-1-1 callers and found that both patients and family members expressed concerns about abnormal, unusual, or severe physical and psychological symptoms- fearing that the symptoms might not subside and potentially lead to a life-threatening situation [9]. This corresponds to the “intensification” described by Laursen et al. [19] and the finding of Mahmuda et al. that when symptoms remain unsolved, concern increases, leading to an emergency call [8].

The findings regarding priority, EMS dispatch and hospital admissions provide important insights into both the EMCC, EMS and the patient population. A recent study by Jamtli et al. [20], conducted at the same EMCC, reported a stroke sensitivity of 77% and a positive predictive value of 16%, but most (68%) of the false positives and false negatives were identified by the EMS in their onsite assessment. In our study, the comparable proportions of Priority 1 cases and hospital admissions in both groups suggest that the EMCC and EMS correctly identify patients with acute or severe conditions. This underscores the presence of genuinely ill or injured patients among the frequent contacts. In addition, 41% of hospital admissions were classified as Priority 2 (urgent) or Priority 3 (non-urgent) by the EMCC. The latter indicates that the EMS appropriately reevaluates the priority while assessing the patient and admits them when needed, with or without conferring with a GP/OOHC doctor. This finding may also reflect the evolving broader scope of the EMS, which extends to facilitating healthcare for patients who require further evaluation despite lower priority or urgency (sub-acute). On the other hand, we also observed that some urgent and acute contacts were resolved without ambulance dispatch. This could be attributed to contacts forwarded to the OOHC for further assessment, alternative care pathways, self-transport, or other arrangements made. While this may indicate efficient triage and resource allocation, it is important to recognize the potential risks associated with this finding, as some urgent or acute cases may have faced delayed intervention or adverse outcomes- that our retrospective dataset would inadvertently miss.

### Future perspectives

While Scott et al. [2] investigated whether the most appropriate threshold was five or six incidents in one month, resulting in 205 patients versus 95 patients, we found almost 20.000 patients using five contacts in one month, over a six-year period. This highlights fundamental differences between the UK and the Norwegian system, making shared definitions and direct comparisons difficult. However, the challenges both the system and patients face, seem very much alike.

Further research is needed to identify effective strategies in the Norwegian context, whether tailored separately for patients with fewer than 12 contacts and those with 12 or more, or to approach both groups combined. An integrative review by Skogevall [21] identified effective strategies for frequent callers and recommend multidisciplinary teams, individualized care plans, and proactive follow-up, because these patients often have complex needs and overlapping challenges, particularly in the areas of mental health, chronic illness, and social instability. They describe how the current situation often is that one engaged individual has taken upon themselves to take on and coordinate a network around these patients. Whereas an approach where a network takes on the patients is a more robust solution, as the success for the individuals often depends on strong collaboration across healthcare services to deliver truly person-centred care. Building on this, a recent review of effective strategies by Jones et al., [22] to reduce ambulance use among frequent callers in the UK point to several promising approaches. They recommend a staggered approach beginning with mass-distribution educational letters, progressing incrementally to structured case management and person-centred care for high-need individuals. As Maruster [10] found, many of those in frequent contact with EMS are also in contact with other healthcare providers, and establishing a clear point of contact for these patients could help reduce the demand on emergency services. It is important to use, and direct patients, to existing services and provide accessible alternatives to the EMCC and EMS. This could include improving same-day access to GPs and enhance public awareness of the OOHC number, particularly for patients with between five and twelve contacts over three months, many of which are likely driven by the need for advice, follow-up, or concerns about worsening symptoms.

All three (10, 21–22) are supportive of using data systems to identify and monitor frequent callers and promoting information sharing across services to manage repeat callers. Escalating this to a system-level and have a cross-sectional network requires EMS administrators and service leaders to connect and collaborate to develop more sustainable and effective service models.

### Recommendation

The ≥ 5 in one month criterion includes many patients who only have a moderate short-term need, whilst also missing some individuals having four contacts per month consecutively. Even though our study was undertaken in just one of Norway’s 16 EMCCs, the Oslo EMCC accounted for 25% of all medical emergency calls. Thus, we suggest committing to just the latter part ≥ 12 in 3 months and excluding ≥ 5 in one month in the Norwegian adaptation of the FreCaNN criteria. This is the exact opposite of what the UK have done in their 2023 update, and we recognise that other services are structured differently and that ≥ 5 in one month is a reasonable threshold. Further studies are needed in services where emergency calls are screened or must be transferred to a medical operator, as preselection is highly likely. Thus, the number of calls placed through might be lower, and the sensitivity and specificity are therefore met at a lower frequency threshold than in Norway.

We also recommend including all ages in the definition, since definitions are used as algorithms for screening. Having to conduct a separate data extraction to discover them is a barrier to recognition, even though the interventions needed likely will differ from the adult population.

### Limitations

As this was a retrospective database study, it included everyone in the database who met the inclusion criteria. We do not know how many contacts were omitted due to an unknown ID, but a recent study on frequent callers contacting EMCC Bergen, Norway, reported 11% missing IDs [13]. Hence, the frequency of both contacts and individuals might be higher. For the included patients and contacts most of the included variables must be filled by the operator, ensuring a high level of completeness and the frequency of “not applicable” values are reported in the tables.

We identified 231 patients under the age of 18 years, and there is a need to address this group separately. In total they accounted for 1.3% of the contacts in the < 12 group and 0.8% in the 12 or more group.

In Table 1, we were unable to determine how the patient was then left on site because ambulance clinicians may first bring the patient to their GP/OOHC for assessment and then continue transporting them to the ED on the same contact. Thus, one cannot just subtract the number of transports from the number of dispatches. Nor do we know the extent of under-triage or how many people who should have had an ambulance dispatched, been seen by a doctor, or been admitted, that were missed.

## Conclusion

One must be aware of the disparities within the group when combining both ≥ 5 individual episodes of care in one month and ≥ 12 episodes in three months in one definition or dataset, as we found the groups to differ in terms of age, gender, caller type, chief complaint, perceived urgency, and response. The ≥ 5-month criterion seems overly sensitive for Norwegian conditions and includes many patients who only have a moderate short-term need. For those with ≥ 12 contacts over three months there is a need to provide accessible alternatives to the EMCC and EMS, and for future research to identify effective targeted interventions.

## Supplementary Information

Below is the link to the electronic supplementary material.

Supplementary Material 1

Supplementary Material 2

Supplementary Material 3

## Data Availability

The data material is available at the Oslo University Hospital secure research server, but restrictions apply to the availability of these data, which were used under the approval from The Norwegian Directory of Health and The Data Protection Official at Oslo University Hospital.
