# Supplementary material for: Disparities between two possible thresholds for frequent contacts to a Norwegian emergency medical communication centre: ≥5 contacts in one month vs. ≥12 contacts in three months
Source: BMC Emerg Med. 2025 Aug 29;25:173. doi: 10.1186/s12873-025-01333-6 (PMC12395745; doi:10.1186/s12873-025-01333-6)
Supplement: Supplementary file 1 — Supplementary Material 1 [file 12873_2025_1333_MOESM1_ESM.docx]

**Supplementary table 1: All sub-categories/criterions for “unidentified problem”**

| **07 Unidentified problem** |
| --- |
| **Criterions** |
| **Priority 1 (acute):** |
| - Does not respond to shaking and shouting - Breathing problems - Still conscious, but sudden faintness / dizziness - Pale and clammy skin, sudden onset - Significantly ill or weak, sudden onset - Unwell and suddenly very frail - Assume serious / critical problem (more information not readily available) - Difficulty communicating, assume serious / critical problem |
| **Priority 2 (urgent):** |
| - Exhausted patient (uncertain / unclear information) - Intense pain - Fainted several times, better now - Assistance required via home alarm system - Functional impairment / rapid decline, recent onset - Assume urgent problem (more information not readily available) - Assume urgent problem (lacking anything applicable in NINM) - Repeated calls, unclear problem - Difficulty communicating, unclear problem |
| **Priority 3 (non-urgent):** |
| - Psychosocial problem - Assume non-urgent problem (more information not readily available) - Assume non-urgent problem (lacking anything applicable in NINM) |

Source: Norwegian Index for Emergency Medical Assistance (Norsk indeks for medisinsk nødhjelp (NIMN)). 2018(4)

<https://www.nakos.no/pluginfile.php/1269/block_html/content/2019%20engelske%20hjelpetekster%20NIMN%204%20nav.pdf>
