# Supplementary material for: Disparities between two possible thresholds for frequent contacts to a Norwegian emergency medical communication centre: ≥5 contacts in one month vs. ≥12 contacts in three months
Source: BMC Emerg Med. 2025 Aug 29;25:173. doi: 10.1186/s12873-025-01333-6 (PMC12395745; doi:10.1186/s12873-025-01333-6)
Supplement: Supplementary file 2 — Supplementary Material 2 [file 12873_2025_1333_MOESM2_ESM.docx]

**Supplementary table 2: Source for variables in dataset**

| **Variables** | **Source** | **Categories** |
| --- | --- | --- |
| Study ID | Aggregated by Oslo University Hospital | Number |
| Year of birth | The Norwegian population registry | YYYY |
| Gender | The Norwegian population registry | Female  Male |
| Type of caller | Set by the EMCC operator | Patient  Next of kin  Neighbour  The public  Police  Fire department  Healthcare personnel  Doctor  OOHC  Other EMCC  Other/NA |
| AMIS problem code | Set by the EMCC operator | 39 categories with sub-criterions^1^ |
| Priority | Set by the EMCC operator | Priority 1 (acute)  Priority 2 (urgent)  Priority 3 (non-urgent) |
| Type of response | Set by the EMCC operator | Ambulance dispatched yes/no  *Note: “no” is the default* |
| Transport destination | Reported to the EMCC by the responding ambulance | No transport  To GP/OOHC  To hospital |

Grey cells were mandatory for study inclusion.

EMCC: Emergency Medical Communication Centre; GP: General practitioner; OOHC: out-of-hours clinic; N/A: Not applicable

1: Norwegian Index for Emergency Medical Assistance (Norsk indeks for medisinsk nødhjelp (NIMN)). 2018(4)

<https://www.nakos.no/pluginfile.php/1269/block_html/content/2019%20engelske%20hjelpetekster%20NIMN%204%20nav.pdf>
