# Supplementary material for: Disparities between two possible thresholds for frequent contacts to a Norwegian emergency medical communication centre: ≥5 contacts in one month vs. ≥12 contacts in three months
Source: BMC Emerg Med. 2025 Aug 29;25:173. doi: 10.1186/s12873-025-01333-6 (PMC12395745; doi:10.1186/s12873-025-01333-6)
Supplement: Supplementary file 3 — Supplementary Material 3 [file 12873_2025_1333_MOESM3_ESM.docx]

|  | **<12 contacts** (p1) | **≥12 contacts** (p2) | Difference  (p2 - p1) | Lower CI | Upper CI |
| --- | --- | --- | --- | --- | --- |
| **Type of caller** | All values are in percentages (%) | | | | |
| Patient | 32 | 64 | 32 | 31.5 | 32.5 |
| Next of kin | 25 | 10 | -15 | -15.4 | -14.6 |
| Healthcare personnel | 23 | 10 | -13 | -13.4 | -12.6 |
| The public | 8.6 | 7.7 | -0.9 | -1.2 | -0.6 |
| OOHC | 3 | 1.4 | -1.6 | -1.8 | -1.4 |
| Doctor | 2 | 0.8 | -1.2 | -1.3 | -1.1 |
| Police | 1.6 | 1.5 | -0.1 | -0.2 | 0 |
| Neighbour | 1.4 | 0.9 | -0.5 | -0.6 | -0.4 |
| Other EMCC | 0.2 | 0.2 | 0 | 0 | 0 |
| Fire department | 0.1 | 0.1 | 0 | 0 | 0 |
|  |  |  |  |  |  |
| **Chief complaint** |  |  |  |  |  |
| Unidentified problem | 22 | 34 | 12 | 11.5 | 12.5 |
| Breathing problems | 11 | 8 | -3 | -3.3 | -2.7 |
| Chest pain | 10 | 7.1 | -2.9 | -3.2 | -2.6 |
| Mental health problems | 8.2 | 20 | 11.8 | 11.4 | 12.2 |
| Abdominal/ back pain | 7.2 | 5.4 | -1.8 | -2.1 | -1.5 |
| Transport | 6.6 | 2.9 | -3.7 | -3.9 | -3.5 |
| Altered level of consciousness* | 5.5 | 2 | -3.5 | -3.7 | -3.3 |
| Intoxication/overdose | 4.4 | 7.2 | 2.8 | 2.6 | 3.1 |
| Minor injuries | 4.1 | 1.9 | -2.2 | -2.4 | -2 |
| Major injuries | 3.5 | 1.5 | -2 | -2.2 | -1.8 |
| Seizures | 1.9 | 1.2 | -0.7 | -0.8 | -0.6 |
| Unresponsive, adult | 1.8 | 1 | -0.8 | -0.9 | -0.7 |
| Urinary tract problems | 1.7 | 1.1 | -0.6 | -0.7 | -0.5 |
| Bleeding, non-traumatic | 1.5 | 0.5 | -1 | -1.1 | -0.9 |
| Fever | 1.4 | 0.5 | -0.9 | -1 | -0.8 |
| Headache | 1.4 | 1.1 | -0.3 | -0.4 | -0.2 |
| Diabetes | 1 | 0.4 | -0.6 | -0.7 | -0.5 |
| Chief complaints <1% | 5 | 2.8 | -2.2 | -2.4 | -2 |
|  |  |  |  |  |  |
| **Priority** |  |  |  |  |  |
| Priority 1, acute | 35 | 18 | -17 | -17.5 | -16.5 |
| Priority 2, urgent | 44 | 32 | -12 | -12.5 | -11.5 |
| Priority 3, non-urgent | 18 | 46 | 28 | 27.5 | 28.5 |
|  |  |  |  |  |  |
| **Response** |  |  |  |  |  |
| Ambulance dispatched | 69 | 38 | -31 | -31.5 | -30.5 |
| Ambulance transport to GP/OOHC | 25 | 17 | -8 | -8.4 | -7.6 |
| Ambulance transport hospital | 32 | 13 | -19 | -19.4 | -18.6 |

**Supplementary Table 2:** **95% confidence interval (CI) for Figure 4, comparing characteristics**
